# Body Mass Index and Dietary Self-Regulation Are Associated with Attenuated Error Monitoring in Young Adults

**DOI:** 10.3390/nu18152545

**Published:** 2026-08-04

**Authors:** Ryan L. Olson, Lisa Qian, Kathryn del Prado, Cody Hamm, Brandon L. Alderman

**Affiliations:** 1 Department of Kinesiology, Health Promotion and Recreation, University of North Texas, Denton, TX 76203, USA; 2 Department of Psychology, University of North Texas, Denton, TX 76203, USA; 3 Department of Kinesiology and Health, Rutgers, The State University of New Jersey, New Brunswick, NJ 08901, USA

**Keywords:** obesity, body mass index, cognitive control, response monitoring, error-related negativity, error positivity, event-related potentials, dietary self-regulation

## Abstract

**Background/Objectives**: Obesity is associated with alterations in cognitive control processes that support performance monitoring and dietary self-regulation, but the neurocognitive markers most closely tied to body weight status in young adulthood remain unclear. **Methods**: Sixty-two young adults completed a modified Simon task while continuous electroencephalography (EEG) was recorded. Response-locked event-related potentials (ERPs) were quantified for error-related negativity (ERN) and error positivity (Pe). The association between body mass index (BMI) and ERP amplitudes was examined under three parameterizations: BMI as a continuous variable, BMI split into three categories (normal weight, overweight, obese), and BMI split into two median-matched categories. **Results**: Higher BMI was associated with reduced (less negative) ERN amplitude under continuous (*r* = 0.30, *p* = 0.016, and median-split, Cohen’s *d* = −0.69, *p* = 0.009) specifications, and at the level of a trend under the three-group classification *F*(2, 59) = 2.80, *p* = 0.069, η^2^p = 0.09. The association was most consistent for neutral trials, where it reached significance under the continuous (*r* = 0.32, *p* = 0.010) and three-group (*F* = 3.31, *p* = 0.043) specifications and approached significance under the median-split (*d* = −0.51, *p* = 0.051). The BMI-ERN association remained significant after adjustment for age, sex, cardiorespiratory fitness, physical activity, and trait impulsivity. In contrast, Pe amplitude did not differ as a function of BMI under any specification. In exploratory analyses that did not survive correction for multiple comparisons, Pe amplitude was positively associated with mindful eating disinhibition and negatively associated with UPPS-P Positive Urgency, independent of BMI. **Conclusions**: These cross-sectional findings indicate that elevated adiposity in young adulthood is associated with attenuated pre-conscious conflict and error monitoring (i.e., ERN), whereas conscious error awareness (i.e., Pe) is unrelated to body weight status. The exploratory Pe–trait associations are offered as hypotheses for future confirmatory work rather than as established effects.

## 1. Introduction

Obesity has emerged as one of the most pressing public health challenges of the twenty-first century. According to the most recent National Health and Nutrition Examination Survey (NHANES) data, the age-adjusted prevalence of adult obesity in the United States is now estimated at 40.3% [1]. Combined overweight and obesity now exceeds 70% of adults [2], a trajectory that shows little sign of abating [3]. Beyond its cardiometabolic effects, obesity is associated with alterations in brain structure and cognitive function, particularly within neural systems involved in executive function and self-regulation [4,5]. Because cognitive control processes shape responses to food cues and guide dietary decisions [6,7], deficits in cognitive control may represent not only a consequence of excess adiposity but also a factor that contributes to the development and maintenance of obesity [8,9].

Cognitive control refers to the top-down, goal-directed processes that coordinate perception, memory, and action [10,11]. Dual-process models of overeating posit that dietary self-regulation reflects a balance between bottom-up appetitive responses and top-down cognitive control processes that constrain impulsive responding [12,13]. Consistent with this framework, individuals with overweight and obesity often exhibit reduced inhibitory control and elevated impulsivity in both general and food-specific contexts [14,15,16], and individual differences in impulsivity prospectively predict future consumption of highly palatable foods [17]. Neuroimaging studies further indicate that obesity is associated with reductions in gray matter volume in the prefrontal cortex (PFC), anterior cingulate cortex (ACC), and orbitofrontal cortex [18,19,20,21], regions critical to performance monitoring and the regulation of motivated behavior, as well as with altered executive control network connectivity during delay discounting and food-related decision making [22].

Although behavioral studies have provided important insights into obesity-related differences in cognitive control, behavioral measures alone may not fully capture alterations in the neural processes that support performance monitoring. Direct empirical support comes from studies of young adults with obesity, in which neuroelectric indices revealed group differences in executive and inhibitory control despite statistically comparable response accuracy and reaction time [23,24,25]. Whereas accuracy and reaction time offer limited information regarding the temporal dynamics of information processing [26,27], event-related brain potentials (ERPs) reflect post-synaptic voltage fluctuations in the continuous electroencephalogram (EEG) that are time-locked to specific sensory, cognitive, or motor events. Their millisecond-level resolution makes ERPs well suited to detecting covert cognitive operations that may be influenced by overweight and obesity even when overt performance remains intact [26,28,29].

Two response-locked ERP components have been instrumental in advancing our understanding of post-response cognitive control: the error-related negativity (ERN) and the error positivity (Pe). The ERN is a negative-going deflection that peaks approximately 50–150 ms after the commission of an erroneous response, with a frontocentral scalp distribution that source-modeling and intracranial work have linked to the supplementary motor area and ACC [30,31]. The ERN is widely interpreted as reflecting the rapid, largely automatic detection of response conflict or the mismatch between an executed response and an intended representation [32,33]. The Pe is a positive-going deflection that follows the ERN, peaks approximately 200–400 ms post-response, and is maximal over centroparietal scalp sites [34]. In contrast to the ERN, the Pe is thought to reflect later, more conscious aspects of error processing, including subjective error awareness, and the recruitment of additional cognitive control resources to adjust behavioral strategy on subsequent trials [34,35]. Together, the ERN and Pe provide functionally distinct indices of early and late stages of performance monitoring and have been shown to be altered in a range of clinical conditions characterized by impaired cognitive control, including externalizing psychopathology [36], major depressive disorder [37], traumatic brain injury [38], and sport-related concussion [39].

Relatively few studies have examined performance-monitoring ERPs in the context of overweight and obesity, and the available findings have been mixed. In a sample of young adults with obesity, Larsen and colleagues [23] observed electrophysiological differences in components related to executive function despite intact behavioral performance, supporting the proposition that neuroelectric measures can detect alterations in cognitive control that are not apparent from response accuracy or reaction time alone. Subsequent work using food and non-food Go/No-Go paradigms has reported reduced P3 amplitudes and slowed responses in obese young adults relative to normal-weight controls, with effects observed for high-calorie food and neutral stimuli alike [24,25].

However, little is known about whether body weight status is associated with neural indices of performance monitoring, such as the ERN and Pe, across the full spectrum of BMI classifications during young adulthood. This represents an important gap because young adulthood represents a developmental period during which obesity-related neurocognitive deficits are detectable before the emergence of more pronounced cognitive or clinical consequences [21,23].

The present study examined the relationship between body weight status and behavioral and neuroelectric indices of performance monitoring during a modified Simon task in young adults. The Simon task reliably elicits response conflict and produces robust ERN and Pe components, making it well suited for examining individual differences in performance monitoring [40,41,42]. We proposed two primary a priori hypotheses. First, consistent with prior behavioral work indicating that young adults with overweight and obesity often show preserved performance on standard cognitive control tasks [14,23], we hypothesized that BMI would be unrelated to response accuracy and reaction time. Second, because neuroelectric measures provide a more sensitive index of subclinical alterations in cognitive control, we hypothesized that higher BMI would be associated with reduced amplitudes of the neuroelectric performance-monitoring components (ERN and/or Pe). Because the ERN and Pe index dissociable stages of error processing [28,34,35], we examined both components without predicting which would prove more sensitive to BMI, allowing the data to clarify whether elevated adiposity is most closely tied to pre-conscious conflict and error monitoring (ERN) or to later evaluative processes (Pe).

To test these hypotheses, we assessed response accuracy, reaction time, ERN amplitude, and Pe amplitude as a function of BMI under three parameterizations: BMI as a continuous variable, BMI split into three clinical categories (normal weight, overweight, obese), and BMI split at the sample-specific median (low, high). Examining multiple parameterizations allowed us to determine whether any observed effects reflected graded dose–response associations with adiposity, categorical thresholds tied to clinical weight classifications, or both. In a set of secondary, exploratory analyses, we further examined whether trait-level measures of mindful eating (MEQ), impulsivity (BIS-11, UPPS-P, I7Q), reward and punishment sensitivity (SPSRQ), and habitual physical activity (IPAQ) were associated with ERN and Pe amplitudes, an approach that allowed us to identify potential cognitive correlates of dietary self-regulation that may operate independently of, or in interaction with, body weight status [8,9].

## 2. Materials and Methods

### 2.1. Experimental Approach

This study used a cross-sectional, correlational design to examine associations between body weight status and behavioral and neuroelectric indices of performance monitoring in young adults. In a single laboratory visit, participants completed the following sets of measurements: (a) anthropometric assessment (height, weight, and BMI) to characterize body weight status; (b) a self-report health history and demographics questionnaire to confirm eligibility and characterize the sample; (c) a battery of validated self-report questionnaires indexing physical activity, mindful eating, dispositional mindfulness, eating behavior, trait impulsivity, and reward and punishment sensitivity as trait-level correlates of dietary self-regulation; (d) a modified Simon task with concurrent EEG to elicit and record response-locked ERPs (ERN and Pe) indexing performance monitoring; and (e) a maximal cardiorespiratory fitness (VO_2_ peak) test as an objective marker of metabolic health. All procedures were approved by the Institutional Review Board of Rutgers, The State University of New Jersey (IRB# E13-041), were conducted in accordance with the Declaration of Helsinki, and were performed in accordance with local and federal regulations. All participants provided written informed consent prior to participation.

### 2.2. Participants

Sixty-two young adults (26 males; 36 females) were recruited from the university community through flyers, classroom announcements, and word-of-mouth referrals. Of the 71 individuals who responded to recruitment and were assessed for eligibility, 6 were excluded prior to testing (4 did not meet inclusion criteria, 2 declined participation) and 3 were excluded following data collection (2 for failing to meet the minimum artifact-free error-trial criterion, 1 for excessive EEG artifact), yielding a final analyzed sample of 62 (see Figure S1 for the participant flow diagram). Eligible participants were between the ages of 18 and 35 years of age (*M* = 20.3, *SD* = 1.6 years), reported normal or corrected-normal vision, and were free from neurological, psychiatric, or cardiovascular conditions. Individuals currently taking psychoactive medications or reporting a history of neurological disorder, psychiatric illness, or head injury involving loss of consciousness were excluded. Additionally, participants were screened via a health history and demographics questionnaire for self-reported metabolic disorders (e.g., diagnosed type 1 or type 2 diabetes, thyroid disorders) and eating disorders. Individuals reporting any such conditions were excluded. Exclusion criteria were verified via participant self-report during initial screening and were re-confirmed by trained laboratory staff upon arrival at the laboratory.

Participants were classified into clinical body mass index (BMI) categories based on standard cutpoints, yielding 21 normal weight (18.5–24.99 kg/m^2^), 22 overweight (25.0–29.99 kg/m^2^), and 19 obese (≥30.0 kg/m^2^) participants. For complementary analyses, participants were also dichotomized at the sample-specific BMI median (27.45 kg/m^2^), yielding 31 low-BMI and 31 high-BMI participants. The median-split was retained only as a secondary robustness check rather than as a primary analysis. Participant demographic data are provided in Table 1.

Using the SPSS effect size specification in G*Power version 3.1.9.7 [43], with 62 participants (normal weight *n* = 21, overweight *n* = 22, obese *n* = 19), α = 0.05, and 80% power, this study had sufficient sensitivity to detect (a) between-group main effects on ERN and Pe amplitudes for the three-group BMI classification at Cohen’s *f* ≥ 0.404 (or partial η^2^ ≥ 0.141); (b) between-group × within-subjects (congruency, accuracy) interaction effects at Cohen’s *f* ≥ 0.202 (or partial η^2^ ≥ 0.039); and (c) within-group effects across the full sample of Cohen’s *d_z_* ≥ 0.362, or within-group effects of Cohen’s *d_z_* ≥ 0.660 for analyses restricted to a single BMI group.

### 2.3. Procedures

Participants completed a single laboratory visit lasting approximately 2 h. All sessions were scheduled between morning to afternoon hours (approximately 9:00 AM to 5:00 PM) to minimize circadian influences on neuroelectric and cognitive performance measures. Participants were instructed to obtain a typical night of sleep the night prior to testing, avoid alcohol consumption for 24 h, refrain from caffeine and other stimulants for 3–4 h, and forgo vigorous-intensity exercise for 24 h prior to arrival. Compliance with these pre-test instructions was verified via self-report at the beginning of the session. No objective biological verification was employed. For female participants, menstrual cycle phase was not systematically recorded. Upon arrival, participants were given a general description of the study, provided written informed consent, and completed a health history and demographics questionnaire to confirm eligibility. The questionnaire assessed age, sex, race, ethnicity, educational attainment, current medication use, tobacco and alcohol use, basic dietary habits, physical activity levels, sleep patterns, and personal history of neurological, psychiatric, cardiovascular, and metabolic conditions. Height and weight were measured using a stadiometer and calibrated digital scale (Health-O-Meter model 499KL, McCook, IL, USA), and BMI was calculated as weight in kilograms divided by height in meters squared (kg/m^2^). Participants then completed the Kaufman Brief Intelligence Test, Second Edition (K-BIT II) [44], which was administered by a trained researcher to provide an estimate of general cognitive ability. Following the K-BIT II, participants completed a battery of self-report questionnaires assessing physical activity, mindful eating, dispositional mindfulness, eating behavior, and trait impulsivity. Participants were then fitted with an EEG sensor net and completed a modified Simon task while continuous EEG was recorded. Following the EEG assessment, participants completed a maximal cardiorespiratory fitness (VO_2_ peak) assessment. Upon completion of the study procedures, participants were debriefed on the purpose of the study.

### 2.4. Questionnaire Battery

The following self-report measures were administered and used in the present analyses. Physical activity was assessed using the International Physical Activity Questionnaire (IPAQ) [45], with total weekly metabolic equivalent (MET)-minutes calculated as the sum of vigorous-, moderate-, and walking-intensity activity. Mindful eating was assessed using the Mindful Eating Questionnaire (MEQ) [46], yielding subscale averages for Awareness, Distraction, Disinhibition, Emotional, and External, with higher scores reflecting greater mindfulness in eating. Dispositional mindfulness was assessed using the Mindful Attention Awareness Scale (MAAS) [47]. Eating behavior was assessed using the Three-Factor Eating Questionnaire-Revised 18 (TFEQ-R18) [48], which provides scores for Uncontrolled Eating, Cognitive Restraint, and Emotional Eating. Trait impulsivity was assessed using three complementary instruments: the Barratt Impulsiveness Scale, Version 11 (BIS-11) [49], which yields first-order (Attention, Cognitive Instability, Motor, Perseverance, Self-Control, Cognitive Complexity) and second-order (Attentional, Motor, Non-Planning) factors and a total impulsivity score; the UPPS-P Impulsive Behavior Scale [50,51], which yields Negative Urgency, Premeditation, Perseverance, Sensation Seeking, and Positive Urgency subscale scores; and the I7 Impulsiveness Questionnaire (I7Q) [52], which yields Impulsiveness, Venturesomeness, and Empathy subscale scores. Sensitivity to reward and sensitivity to punishment were assessed using the Sensitivity to Punishment and Sensitivity to Reward Questionnaire (SPSRQ) [53]. All questionnaires were scored according to published guidelines.

### 2.5. Cardiorespiratory Fitness (VO_2_ Peak) Assessment

VO_2_ peak was assessed using a continuous graded maximal exercise test on a cycle ergometer (Lode Corival, Groningen, NLD). Following a 3 min warm-up at 50 W and ≥60 rpm, workload was increased by 25 W every 2 min until volitional exhaustion or the inability to maintain the prescribed pedaling cadence (≥60 rpm) occurred. Expired gases were measured continuously using an open-circuit indirect calorimetry system (Parvo Medics TrueOne 2400, Sandy, UT, USA). Heart rate and rating of perceived exertion (Borg’s 6–20 scale) were monitored throughout the test. VO_2_ peak was defined as the highest 15 s average oxygen uptake observed during the test, provided at least two of the following criteria were met: respiratory exchange ratio ≥ 1.10, peak heart rate within 10 beats per minute of age-predicted maximum, or plateau in VO_2_ despite increasing workload. Following completion of the test, a 3–5 min cool-down was provided at a self-selected resistance and pedaling cadence.

### 2.6. Simon Task

A modified Simon task [40] was administered using E-Prime Professional version 2.0 software (Psychology Software Tools, Inc., Pittsburgh, PA, USA), with the computer monitor positioned at a distance of approximately 70 cm from the participant, centered to the nasion. On each trial, participants were presented with a colored arrow (3.5 cm × 3.5 cm) that appeared in the center of the screen and were asked to respond to the color of the arrow (green or red) by pressing the corresponding left or right trigger, irrespective of the arrow’s pointed direction. Stimulus–response mappings (color-to-hand) were counterbalanced across participants. Trials were classified as congruent when the color of the arrow matched the response side (e.g., a green arrow pointing to the right with a right-hand response mapping), incongruent when the color of the arrow conflicted with the response side (e.g., a green arrow pointing left with a right-hand response mapping), or neutral when the color of the arrow neither matched nor conflicted with the response side (e.g., a green arrow pointing up with a right-hand response mapping). Each trial began with a white fixation cross (+) presented for 500 ms on a black background, followed by the colored arrow stimulus presented for 100 ms. Participants were given 1000 ms to respond, followed by a random inter-trial interval of 900–1300 ms. Following verbal and on-screen instructions emphasizing both speed and accuracy, participants completed 20 practice trials with performance feedback before completing the experimental task. The experimental task consisted of six blocks of 96 randomly ordered and equiprobable congruent, incongruent, and neutral trials, separated by 3 min rest periods, yielding 576 total trials.

### 2.7. EEG Acquisition

Continuous EEG, referenced to the vertex electrode (Cz), was recorded from 64 scalp electrode sites using an EGI HydroCel Geodesic Sensor Net (Electrical Geodesics, Inc., Eugene, OR, USA) arranged based on the International 10-10 system [54]. The data were amplified and digitized using an EGI amplifier with a nominal gain of 20,000, an online bandpass filter of 0.10–100 Hz, and a sampling rate of 250 Hz with a 24-bit analog-to-digital converter. Horizontal electrooculogram (EOG) activity was recorded from electrodes located lateral to the external canthi to measure saccadic eye movements, while vertical EOG activity was recorded from electrodes located above and below each eye to measure blinks. Continuous data were visualized in Net Station 4.0 during acquisition, and electrode impedances were maintained below 50 kΩ [55] prior to and during the recording. EEG recording was conducted in a dimly lit laboratory maintained at a consistent ambient temperature (approximately 21–23 °C) with minimal environmental noise. Participants were seated in a comfortable chair approximately 70 cm from the stimulus display and were instructed to minimize head and body movement during task performance.

### 2.8. EEG Data Processing

All data were exported from Net Station 4.0 to the EEGLAB toolbox [56] in MATLAB R2025a (MathWorks, Natick, MA, USA) to perform data preprocessing operations. Data were bandpass filtered using a 2nd-order infinite impulse response (IIR) Butterworth filter of 0.1–30 Hz. Data were then adjusted for direct current (DC) offset and visually inspected to identify and remove segments containing large muscle-related artifacts or extreme offsets. Response-locked epochs were created for both correct and incorrect (error commission) trials using a −200 to 800 ms time window relative to the participant’s response. Ocular artifacts and eye blinks were removed from the segmented waveforms using independent component analysis (ICA) blink templates provided by the ERP PCA Toolkit [57] and generated from the current dataset. ICA components that correlated 0.9 or higher with the scalp topographies of the blink template were removed. Across the sample, an average of 2.8 (*SD* = 1.4) independent components were identified as artifactual and removed per participant. Trials were rejected if there was a voltage difference of more than 100 μV between the minimum and maximum values within the trial, or if individual channels differed by more than 30 μV from the six closest neighboring channels. Trials with more than 10% of channels marked as bad were also removed. The mean percentage of rejected trials per participant was 8.3% (*SD* = 4.1%). Channels exhibiting persistent excessive noise, identified through visual inspection and the listed criteria above, were interpolated using the average of neighboring channels weighted by their geodesic distance. An average of 1.6 (*SD* = 1.3) noisy channels per participant were identified and interpolated. Three individuals were excluded prior to statistical analysis for failing to meet the six-error-trial minimum (*n* = 2) or for excessive artifact (*n* = 1). The mean number of accepted error trials available for ERN and Pe quantification was 34.6 (*SD* = 11.2) per participant while the mean number of accepted correct trials exceeded 300 per participant. The average number of accepted correct trials per participant was 136.6 (*SD* = 15.8) for neutral, 141.0 (*SD* = 14.5) for congruent, and 127.4 (SD = 19.0) for incongruent conditions while the average number of accepted error trials was 14.4 (*SD* = 6.2) for neutral, 11.3 (*SD* = 4.9) for congruent, and 23.7 (*SD* = 8.1) for incongruent conditions. Following artifact removal, data were re-referenced to averaged mastoids [58,59], averaged separately by trial type (congruent, incongruent, neutral) and response accuracy (correct, error), and baseline-corrected using the −200 to 0 ms pre-response period. Consistent with prior recommendations for stable response-locked ERP estimation [60], participants were required to contribute a minimum of 6 artifact-free error trials for ERN and Pe quantification.

### 2.9. ERP Quantification

Consistent with previous ERP research on response monitoring and based on the scalp distributions of the components of interest, the error-related negativity (ERN) was quantified across a region of interest (ROI) at frontocentral electrode sites (EGI: 4, 7, 54) [34,61], and the error positivity (Pe) was quantified across a centroparietal ROI (EGI: 33, 34, 36, 38) [35,62]. The ERN was measured as the mean amplitude in an a priori time window of 50–150 ms post-response on incorrect trials, while the Pe was measured as the mean amplitude in an a priori time window of 200–400 ms post-response on incorrect trials [34,39]. Mean amplitudes were extracted separately for each trial type (congruent, incongruent, neutral) to evaluate congruency effects on the ERN and Pe components. Absolute ERN and Pe amplitudes on error trials were used as the primary outcomes rather than error-minus-correct difference scores because (a) the study hypotheses concerned the amplitude of error-related brain activity itself in relation to BMI, rather than the relative difference between response types, (b) error-trial amplitudes directly index the neural response to error commission and are not subject to the reduced reliability that can arise from subtracting the correct-trial response, which itself reflects meaningful monitoring activity, and (c) recent psychometric work indicates that absolute error-related amplitudes typically show comparable or superior internal consistency to difference scores in adult samples. We nonetheless recognize that error-minus-correct difference scores can control for overlapping non-error activity. Correct-related negativity (CRN) values were quantified for descriptive comparison but were not the focus of the primary analyses.

### 2.10. Data Analysis

Data were analyzed using SPSS version 29 (IBM Corp., Armonk, NY, USA). Analyses were organized a priori into primary (confirmatory) and secondary (exploratory) families. The primary analyses tested the association between BMI and ERN/Pe amplitudes while the secondary, exploratory analyses comprised the correlations between the questionnaire battery and ERP amplitudes. To characterize the relationship between body weight status and ERP indices of performance monitoring, the BMI-ERP association was examined under three parameterizations of weight status. First, BMI was treated as a continuous predictor, and Pearson correlations and simple linear regression models were used to estimate the strength and direction of the BMI-ERP association across the full sample (*N* = 62). Second, participants were classified into three standard clinical BMI categories (normal weight, overweight, obese), and one-way analyses of variance (ANOVAs) were used to test for group differences in ERN amplitude, Pe amplitude, response accuracy, and reaction time. Significant omnibus effects were followed by Tukey’s Honestly Significant Difference (HSD) post hoc tests. Third, participants were split into two BMI categories at the sample-specific median (cutpoint = 27.45 kg/m^2^), and independent-samples *t*-tests with Cohen’s *d* as the effect size estimate were used to compare low- and high-BMI participants on each outcome. The continuous specification was treated as the primary test of the BMI-ERP association because it retains the full information in the BMI distribution and does not depend on categorical cutpoints. The three-category (clinical BMI) parameterization was included because it aligns with the standard clinical framework used to communicate obesity-related findings. The median-split parameterization was included as a robustness check to ensure that the primary continuous findings were not obscured by nonlinearity in the middle of the BMI distribution. We recognize that dichotomization at the median reduces statistical power, discards information contained in the continuous variable, and is generally discouraged in the methodological literature [63]. Accordingly, the median-split was not treated as a primary analysis. Instead, it was retained solely as a transparent robustness check to determine whether effects observed with continuous BMI were also detectable under a simple categorical contrast, alongside the clinically meaningful three-group classification. Convergence across the continuous, three-group, and median-split parameterizations was interpreted as evidence that findings were not artifacts of a single analytic decision, whereas any divergence was interpreted with corresponding caution.

Behavioral congruency effects (congruent vs. incongruent trials) were tested with repeated-measures ANOVAs that included BMI category (3-level) or BMI median-split (2-level) as a between-subjects factor. ERN and Pe amplitudes were extracted for neutral, congruent, and incongruent trials, and the same model structure was applied to the ERP data. To evaluate the robustness of the primary BMI-ERP associations to potential confounders, multiple linear regression models were estimated in which ERN and Pe amplitudes were regressed on BMI together with age, sex, VO_2_ peak, physical activity (IPAQ total MET-minutes), and trait impulsivity (BIS-11 total). To examine whether trait-level individual differences in mindful eating, impulsivity, and physical activity were related to performance monitoring independent of body weight, Pearson correlations between each questionnaire subscale and each ERP outcome were computed across the full sample. Significant trait-ERP associations were further examined in joint regression models that included BMI as a covariate to determine whether the trait-ERP relationship persisted after accounting for body weight status. Because the questionnaire-ERP correlations were exploratory and numerous, *p*-values within this family were adjusted using the Benjamini–Hochberg false discovery rate (FDR) procedure [64], and both uncorrected and FDR-adjusted *p*-values are reported. These associations are interpreted as hypothesis-generating rather than confirmatory. Missing questionnaire responses were minimal (<2% overall) and were handled via listwise deletion within each analysis. No participant had missing data on the primary ERP or anthropometric variables.

Prior to analysis, the assumptions underlying parametric tests were evaluated. Normality was assessed using Shapiro–Wilk tests and inspection of distributions, homogeneity of variance across BMI groups was assessed using Levene’s test, and univariate outliers were screened using standardized scores (*z* > 3.29). The primary ERP outcomes (ERN and Pe) were normally distributed with homogeneous variances and no extreme outliers. Behavioral outcomes (accuracy and reaction time) departed from normality; thus, the corresponding associations were confirmed using nonparametric (Spearman) correlations. Greenhouse–Geisser corrections were applied where appropriate to repeated-measures effects with more than two levels, and partial eta-squared (ηp^2^) and Cohen’s *d* are reported as effect size estimates. All analyses were conducted with α = 0.05 (two-tailed). Effect sizes (ηp^2^, Cohen’s *d*, and *r*) are reported alongside all test statistics to support interpretation.

## 3. Results

Assumption checks supported the use of parametric tests for the primary outcomes: ERN and Pe amplitudes were normally distributed (Shapiro–Wilk *p*s > 0.05) with homogeneous variances across BMI groups (Levene’s *p*s > 0.22) and no univariate outliers (*z* < 3.29). Accuracy and reaction time departed from normality and were therefore additionally examined with Spearman correlations, which yielded conclusions identical to the parametric analyses. BMI groups did not significantly differ in age, height, or estimated IQ (*p*s > 0.05). As expected, significant between-group differences were observed for weight, BMI, and VO_2_ peak. VO_2_ peak declined in a graded fashion across BMI categories, *F*(2, 59) = 8.63, *p* < 0.001, η^2^p = 0.23, and was negatively correlated with BMI when modeled continuously (*r* = −0.44, *p* < 0.001); group means are reported in Table 1. Self-reported physical activity followed the same direction, with a lower IPAQ total in the obese group that approached significance, *F*(2, 59) = 2.62, *p* = 0.081, and a significant negative correlation with continuous BMI (*r* = −0.32, *p* = 0.010). VO_2_ peak and IPAQ were positively correlated (*r* = 0.41, *p* = 0.001), confirming convergence between the objective and self-report activity measures. Trait impulsivity also varied by BMI group, with higher BIS-11 total scores in overweight and obese participants relative to normal weight participants, *F*(2, 59) = 4.04, *p* = 0.023, and a comparable pattern for the BIS Self-Control/Cognitive Complexity second-order factor, *F*(2, 59) = 4.28, *p* = 0.018.

### 3.1. Behavioral Performance

#### 3.1.1. Response Accuracy

**Figure 1:**
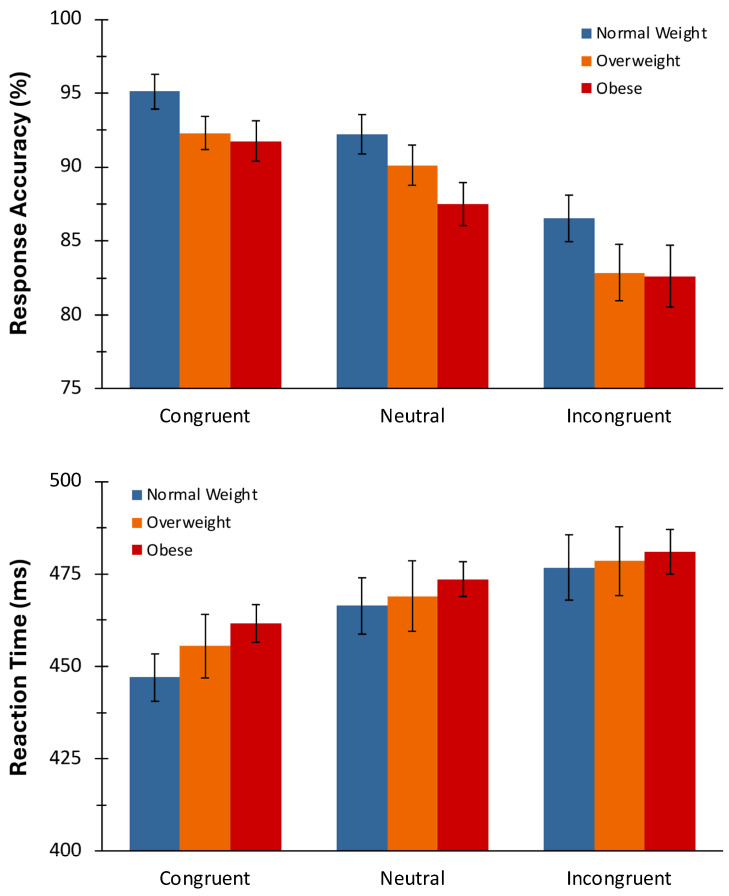
Average response accuracy (**top**) and reaction time (**bottom**) from the Simon Task measured in normal weight, overweight, and obese individuals.

As expected, participants demonstrated higher accuracy on congruent (*M* = 93.4%) relative to incongruent (*M* = 84.0%) trials, confirming the standard congruency effect. With BMI treated continuously, higher BMI was associated with lower overall response accuracy (*r* = −0.25, *p* = 0.048) and with lower accuracy specifically on neutral (*r* = −0.32, *p* = 0.012) and congruent (*r* = −0.26, *p* = 0.039) trials. Under the three-group classification, the BMI main effect on overall accuracy did not reach significance, *F*(2, 59) = 2.25, *p* = 0.114 although the pattern was directionally consistent with the continuous analysis (Figure 1). Under the median-split, accuracy was lower in high-BMI than in low-BMI participants on neutral trials, *t*(60) = 2.23, *p* = 0.029, Cohen’s *d* = 0.57, and at the level of an overall trend, *t*(60) = 1.84, *p* = 0.071, *d* = 0.47. Taken together, accuracy effects emerged most clearly under the continuous parameterization.

#### 3.1.2. Reaction Time

As expected, participants responded more slowly on incongruent (*M* = 478.7 ms) than congruent (*M* = 454.5 ms) trials. BMI was not significantly associated with overall reaction time under any of the three parameterizations (continuous *r* = 0.09, *p* = 0.50; three-group *F*(2, 59) = 0.30, *p* = 0.74; two-group *t*(60) = −1.63, *p* = 0.11). Under the median-split, congruent-trial reaction time was significantly slower in the high-BMI group than in the low-BMI group, *t*(60) = −2.01, *p* = 0.041, Cohen’s *d* = −0.51, although this trial-type-specific effect did not reach significance under the continuous (*r* = 0.17, *p* = 0.19) or three-group (*F*(2, 59) = 1.04, *p* = 0.36) parameterizations and should be interpreted cautiously (Figure 1). In contrast to the limited BMI–reaction time association, cardiorespiratory fitness was negatively associated with reaction time across all trial types. Higher VO_2_ peak predicted faster overall reaction time (*r* = −0.27, *p* = 0.036), as well as faster reaction time on neutral (*r* = −0.26, *p* = 0.040), congruent (*r* = −0.27, *p* = 0.037), and incongruent (*r* = −0.25, *p* = 0.047) trials. The VO_2_ peak–reaction time association remained significant after controlling for BMI in a joint regression model, β = −1.02 ms per mL/kg/min, *p* = 0.049, with β BMI = −0.22 ms per BMI unit, *p* = 0.82. Response accuracy was not associated with VO_2_ peak (overall *r* = 0.08, *p* = 0.52).

### 3.2. ERP Results

#### 3.2.1. Error-Related Negativity (ERN)

**Figure 2:**
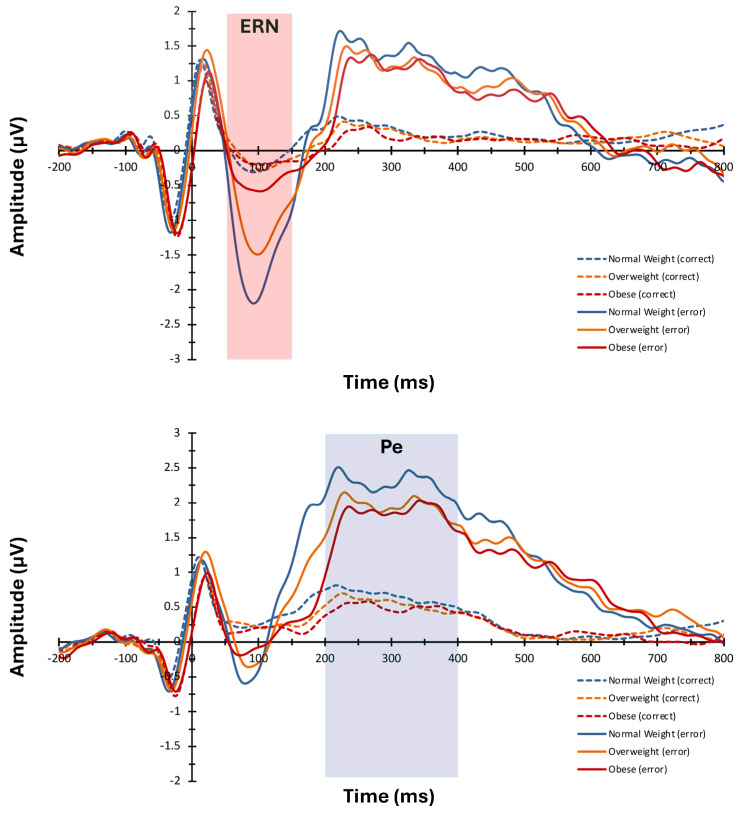
Grand average ERN (**top**) and Pe (**bottom**) waveforms averaged across frontocentral and centroparietal electrode sites for normal weight, overweight, and obese individuals.

Higher BMI was associated with reduced (less negative) ERN amplitude. When BMI was modeled continuously, higher BMI predicted a less negative ERN overall (*r* = 0.30, *p* = 0.016; β = 0.065 μV per BMI unit) and on neutral and incongruent trials, with the congruent-trial association falling just short of significance. Under the three-group classification, the overall main effect was marginal (*p* = 0.069) and reached significance only on neutral trials (*p* = 0.043). Statistics for all parameterizations and trial types are reported in Table 2 while group waveforms are shown in Figure 2. Tukey HSD post hoc comparisons indicated that the normal weight and obese groups differed significantly on neutral-trial ERN amplitude (mean difference = 0.61 μV, *p* = 0.037), while the normal weight–overweight (*p* = 0.239) and overweight–obese (*p* = 0.599) contrasts did not reach significance. The BMI-ERN association was robust to adjustment for covariates. In a multiple regression model including age, sex, VO_2_ peak, physical activity, and trait impulsivity (BIS-11 total), BMI remained a significant predictor of overall ERN amplitude (*b* = 0.072 μV per BMI unit, *t*(55) = 2.32, *p* = 0.024) and of neutral-trial ERN amplitude (*b* = 0.071, *t*(55) = 3.05, *p* = 0.004); sex was the only additional significant predictor of overall ERN amplitude (*p* = 0.046), and neither VO_2_ peak nor physical activity was a significant predictor (*p*s > 0.60). The association was likewise unchanged when BMI was modeled with Spearman rank correlations (overall *r_s_* = 0.32, *p* = 0.013; neutral *r_s_* = 0.32, *p* = 0.010).

**Figure 3:**
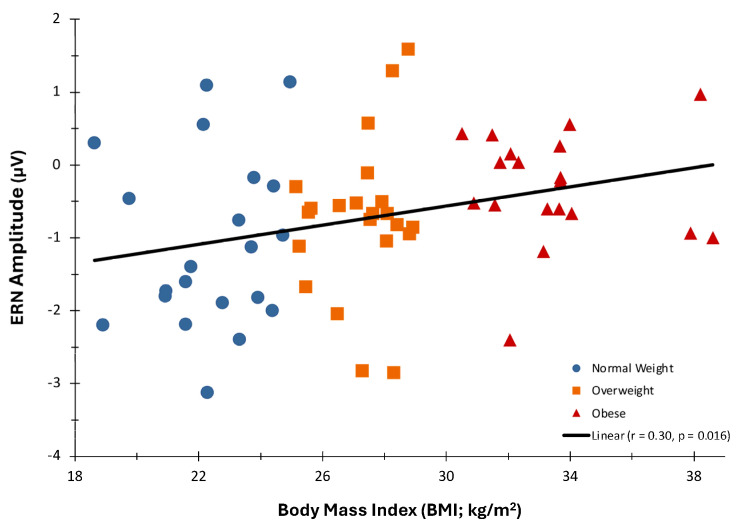
Association between BMI and ERN amplitude.

Under the median-split, low-BMI participants showed significantly more negative ERN amplitudes than high-BMI participants overall and on incongruent trials, with the neutral-trial effect at the threshold of significance and the congruent-trial effect marginal (Table 2). Taken together, the BMI–ERN association is supported most consistently by the continuous and median-split overall analyses and by the neutral-trial three-group analysis (Figure 3).

#### 3.2.2. Error Positivity (Pe)

In contrast to the ERN findings, Pe amplitude was not associated with BMI (Figure 2). The continuous, three-group, and median-split tests were all non-significant, as were the corresponding trial-type-specific tests (all *p*s > 0.26; Table 2). Thus, BMI-related differences in performance monitoring appeared specific to the ERN component.

### 3.3. ERP Individual Differences Associations

**Figure 4:**
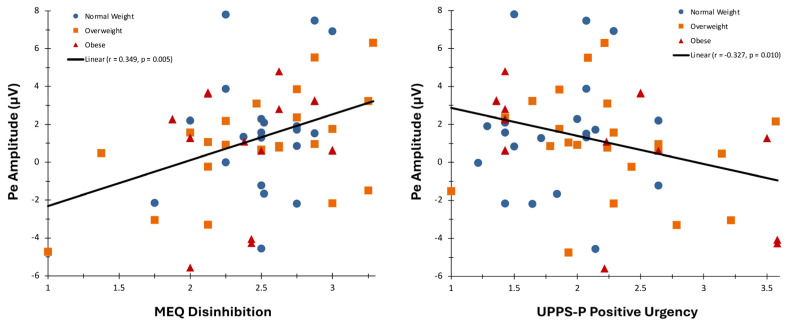
Associations between Pe amplitude and mindful eating–disinhibition scores (**left**), and Pe amplitude and UPPS-P Urgency scores (**right**).

In a set of exploratory analyses of the questionnaire battery, Pe amplitude was related to trait-level individual differences in mindful eating and impulsivity in directions consistent with dietary self-regulation (Figure 4). Higher scores on the MEQ Disinhibition subscale (i.e., less disinhibited eating) were associated with larger overall Pe amplitude (*r* = 0.35, *p* = 0.006). Higher scores on the UPPS-P Positive Urgency subscale (i.e., greater tendency to act rashly under positive affect) were associated with smaller overall Pe amplitude (*r* = −0.33, *p* = 0.009). These associations were significant at the uncorrected threshold and remained significant after controlling for BMI in joint linear regression models (MEQ Disinhibition, β = 2.39, *t*(59) = 2.81, *p* = 0.007, β BMI = −0.04, *p* = 0.60; UPPS-P Positive Urgency, β = −1.46, *t*(59) = −2.58, *p* = 0.012, β BMI = −0.02, *p* = 0.81). However, neither association survived Benjamini–Hochberg FDR correction across the exploratory questionnaire family (both *p_FDR_* = 0.10), and both are therefore interpreted as hypothesis-generating rather than as robust effects. No questionnaire measure was associated with ERN amplitude after FDR correction (all *p_FDR_* > 0.10).

## 4. Discussion

The primary aim of the present study was to examine the relationship between body weight status and behavioral and neuroelectric indices of performance monitoring during a modified Simon task in young adults. The main finding was that higher BMI was associated with reduced (less negative) ERN amplitude, whereas Pe amplitude was unrelated to BMI. This pattern was observed despite relatively modest behavioral differences and suggests that elevated adiposity may be associated with alterations in early-stage performance monitoring that are more readily detected using neuroelectric measures than conventional behavioral outcomes. In exploratory analyses, Pe amplitude was associated with individual differences in mindful eating and Positive Urgency independent of BMI. However, these associations did not survive correction for multiple comparisons, thus they are discussed below as preliminary and hypothesis-generating. Because the present design was cross-sectional, all associations reported are correlational and do not establish that elevated adiposity causes altered error monitoring or vice versa.

The association between higher BMI and reduced ERN amplitude is consistent with the broader literature suggesting that ERPs may be sensitive to subtle alterations in cognitive control that are not readily apparent in behavioral performance alone [23,28]. Several recent studies have reported electrophysiological differences in young adults with obesity despite largely intact reaction time and accuracy on standard cognitive control tasks [23,24,25]. However, the broader ERP literature on obesity and cognitive control remains mixed, with some studies reporting altered components and others reporting null or inconsistent effects [23,24,25,65]. The present ERN finding should therefore be weighed against this heterogeneity rather than treated as a settled effect. The current findings extend this body of work by demonstrating that elevated adiposity is associated with attenuation of pre-conscious error monitoring during a Simon task, a paradigm with well-established stimulus–response conflict properties that, to our knowledge, has not previously been used to compare ERN amplitudes across the full range of body weight categories in young adulthood. The convergence of findings across continuous, three-group, and median-split parameterizations of BMI provides confidence that the BMI-ERN association is not an artifact of any single categorical decision but reflects a graded relationship between adiposity and pre-conscious conflict and error monitoring. This association also remained significant after adjustment for age, sex, cardiorespiratory fitness, physical activity, and trait impulsivity, indicating that it is not readily attributable to these covariates. The graded BMI-related decrement in response accuracy observed under the continuous specification further suggests that behavioral differences in cognitive control may emerge alongside the neuroelectric differences but are detected more reliably by ERP measures than by behavioral measures of comparable trial counts.

Because the ERN and Pe are widely interpreted as functionally dissociable components of performance monitoring [34,35,36], the selective reduction in ERN amplitude alongside preserved Pe suggests that young adults with elevated adiposity exhibit attenuated early-stage conflict and error detection at the neural level while retaining the capacity to engage downstream evaluative processes. A reduction in ERN amplitude has been reported in conditions characterized by impaired top-down cognitive control, including externalizing psychopathology [36] and traumatic brain injury [38], where it has been interpreted as a marker of attenuated activity in the anterior cingulate cortex (ACC) and supplementary motor regions of the medial prefrontal cortex that contribute to performance monitoring [32,33]. Whether elevated adiposity is accompanied by comparable differences in these circuits cannot be determined from the present data. The study did not include structural or functional neuroimaging, and scalp-recorded ERPs do not permit localization of their underlying neural generators. Possible explanations of the attenuated ERN, including reduced rapid conflict detection, altered ACC-mediated error processing, or diminished prediction-error signaling, are therefore offered as hypotheses consistent with the prior imaging literature rather than as mechanisms the present design can test.

The reduced ERN amplitudes observed in higher-BMI participants are consistent with a growing body of work linking elevated adiposity to structural and functional alterations in frontocingulate networks. Voxel-based morphometric studies have reported reduced gray matter volume in the prefrontal, anterior cingulate, and orbitofrontal cortices among individuals with overweight and obesity [18,19,20], regions that have been implicated as proposed medial frontal generators of the ERN and as part of the broader performance-monitoring network [32,33]. Functional neuroimaging work has further demonstrated altered executive control network connectivity and impaired delay discounting performance in individuals with obesity, consistent with the proposition that obesity is associated with attenuated top-down regulation of motivated behavior [22]. The present finding of reduced ERN amplitude with elevated adiposity provides converging electrophysiological evidence for this proposition and is compatible with the possibility that frontocingulate contributions to performance monitoring are altered in young adulthood, well before the emergence of overt cognitive decline. As with all scalp-recorded ERP work, the specific neural generators of these effects cannot be determined from the present data alone and require confirmation with source-localized or multimodal imaging approaches. Trait impulsivity also differed across BMI categories in the present sample, although BIS-11 total did not statistically mediate the BMI-ERN association. This pattern may reflect the broader notion that elevated impulsivity and reduced cognitive control co-occur with elevated adiposity but represent partially independent contributions to dysregulated eating behavior.

Cardiorespiratory fitness showed a distinct effect: VO_2_ peak tracked reaction time across all trial types, even after controlling for BMI, but was unrelated to ERN and Pe amplitude. Different facets of metabolic health therefore appear to track different cognitive control outcomes. Body weight status appears to selectively track pre-conscious conflict and error monitoring (ERN), while cardiorespiratory fitness appears to track general processing speed (reaction time) rather than the neuroelectric performance monitoring components. This dissociation is broadly consistent with the broader exercise-cognition literature that links cardiorespiratory fitness to faster response speeds and enhanced cognitive control in young adults [28,29,66], and it adds further specificity to the present finding by indicating that the BMI-ERN association is not simply attributable to fitness-related variation. From a translational standpoint, the convergent reductions in fitness and pre-conscious error monitoring with elevated adiposity suggest that interventions targeting both adiposity and cardiorespiratory fitness, such as structured aerobic exercise programs, may have additive cognitive benefits.

Although the following trait associations did not survive correction for multiple comparisons and are therefore preliminary, their direction is theoretically coherent and may guide future confirmatory work. Larger Pe amplitudes in participants reporting less disinhibited eating and lower Positive Urgency are consistent with dual-process accounts of overeating, which hold that dietary self-regulation reflects a balance between appetitive responses to food cues and top-down cognitive control [12,13], and that food-related and general impulsivity, particularly the tendency to act rashly under positive affect, are strong predictors of overconsumption and future weight gain [15,17]. The Pe is well-positioned to track the moment-to-moment recruitment of cognitive resources required to consciously evaluate and adjust behavior when food choices conflict with longer-term dietary goals, and the present results tentatively suggest that trait-level individual differences in this evaluative process align with self-reported dispositional dietary self-regulation. Combined with the BMI-related reduction in ERN, this pattern provides preliminary evidence that pre-conscious conflict and error monitoring (ERN) and conscious error awareness (Pe) may index partially distinct cognitive pathways relevant to dietary behavior: ERN appears to track current weight status, whereas Pe appears to track trait-level dispositional dietary self-regulation independent of weight.

The robust congruency effects observed for accuracy and reaction time replicate prior work with the Simon and related conflict paradigms [41,42] and indicate that the task elicited the expected stimulus–response conflict, so the BMI-related ERN effect cannot be attributed to a failure of the paradigm to engage cognitive control. For the ERN, the BMI association was strongest on neutral trials and somewhat attenuated on congruent and incongruent trials, where the three-group main effects reached only marginal significance; this pattern is consistent with the interpretation that BMI-related reductions in ERN reflect a general decrement in pre-conscious conflict and error monitoring rather than a trial-type-specific deficit, and may also reflect the higher signal-to-noise ratio typically observed on neutral trials in this paradigm. For the Pe, no BMI-related differences emerged at any trial type, reinforcing the conclusion that conscious error evaluation is preserved across the BMI range in this sample of young adults.

Several limitations of the present study warrant consideration. First, BMI is an indirect measure of adiposity and does not distinguish between fat mass, lean mass, and the regional distribution of body fat. More direct measures of adiposity, such as dual-energy X-ray absorptiometry (DEXA) or measures of visceral adipose tissue, may provide additional specificity in linking adiposity to neuroelectric indices of cognitive control [16]. Second, the present sample consisted of college-age young adults, and the generalizability of these findings to older adults, clinical samples, and individuals with severe obesity remains to be established. Recent work suggests that the relationship between elevated adiposity and executive function may be most pronounced at higher BMI strata, including class II and class III obesity [14], and that obesity-related cognitive vulnerability may compound with age [21]. Third, although the present study included a broad battery of widely used self-report measures of trait impulsivity, mindful eating, and reward sensitivity, the field has continued to refine more food-specific and behaviorally validated measures of impulse control, attentional bias, and reward sensitivity [12,15,17], which may be more directly relevant to dietary self-regulation. Fourth, the cross-sectional design precludes inference about the directionality of the association between body weight status and ERN amplitude; longitudinal work is needed to determine whether reductions in ERN amplitude precede, accompany, or follow the development of overweight and obesity. Fifth, the present sample provided sufficient power to detect medium-to-large effects under the planned BMI parameterizations but was modest for detecting the smaller trial-type-specific Pe effects or higher-order interactions. Replication in larger samples will be required to characterize subtle differences in conscious error monitoring. Relatedly, statistical power was limited for detecting small-to-moderate effects and for subgroup analyses. The median-split and three-group contrasts should accordingly be regarded as secondary, as the effect size estimates may be unstable in a sample of this size. Sixth, adherence to the pre-test instructions (sleep, exercise, caffeine, and alcohol restrictions) lacked objective confirmation and was verified only by self-report, so a lack of adherence cannot be excluded. Seventh, we did not record the precise time of day of testing beyond the standardized daytime window, participants’ fasting or feeding status, prior-night sleep duration, recent alcohol consumption beyond the 24 h restriction, or menstrual cycle phase. These factors may introduce variability into ERP measures and should be considered in future work. Finally, the present analyses did not include direct measures of eating behavior or food intake. As a result, the inferential link between the present neuroelectric findings and actual dietary behavior remains indirect, and we caution against interpreting the ERP associations as direct markers of eating behavior. Pairing ERP measures with validated dietary assessments and food-cue paradigms will be an important next step.

Several directions for future research follow from these limitations and findings. Studies that pair more precise measures of adiposity with ERP indices of pre- and post-response cognitive control would help to clarify the extent to which the present BMI-ERN association reflects total adiposity, regional fat distribution, or related cardiometabolic factors. Investigations that incorporate food-specific cognitive control paradigms, such as food go/no-go or food Stroop tasks, alongside non-food paradigms may help to determine whether the reduced ERN observed here generalizes to or interacts with appetitive food cues [24,25], and whether the trait-level Pe associations observed for mindful eating and Positive Urgency are amplified when stimulus context is dietarily relevant. Longitudinal designs that follow young adults across the transition into middle age would help to establish the temporal sequencing of obesity-related changes in ERN amplitude and behavioral performance, and to clarify whether attenuated ERN amplitude precedes, accompanies, or follows the development of overweight and obesity. Intervention studies, including food-related response inhibition training [67,68], aerobic exercise [28,66], and structured dietary interventions, could determine whether the BMI-ERN association is modifiable and whether changes in ERN amplitude predict sustained changes in eating behavior. Consistent with calls for methodological rigor and replication in this area, pre-registered designs, standardized ERP processing pipelines [27], and adequately powered samples will be essential to disentangle the cognitive determinants of dietary behavior from their behavioral correlates. Finally, integration of ERP indices with other neuroscientific methods (e.g., structural and functional MRI, gut microbiome characterization) may help to identify the neural and physiological pathways through which elevated adiposity influences performance monitoring [4,5,65].

## 5. Conclusions

In a sample of 62 young adults examined under three complementary parameterizations of body weight status, higher BMI was robustly associated with reduced (less negative) ERN amplitude during a modified Simon task, with effect sizes in the medium-to-large range across continuous, three-group, and median-split specifications, and this association persisted after adjustment for age, sex, cardiorespiratory fitness, physical activity, and trait impulsivity. Pe amplitude did not vary as a function of BMI. In exploratory analyses that did not survive correction for multiple comparisons, Pe amplitude was instead related to trait mindful eating disinhibition and Positive Urgency, independent of BMI. Because the design was cross-sectional, these findings are associational; they generate, rather than confirm, the hypothesis that pre-conscious (ERN) and conscious (Pe) performance monitoring may index partially distinct pathways relevant to dietary behavior, with ERN more closely tracking current weight status and Pe more closely tracking trait dietary self-regulation. Testing this hypothesis will require adequately powered, pre-registered, and longitudinal studies. Within the broader effort to characterize the cognitive determinants of dietary behavior, the present results highlight the value of pairing neuroelectric measures of performance monitoring with multiple parameterizations of body weight status and validated individual-difference measures, and suggest that both weight-tracking and trait-tracking neuroelectric markers may help to identify individuals whose cognitive profile renders them more vulnerable to lapses in dietary self-regulation and who may therefore benefit most from cognition-focused dietary interventions. Future research that incorporates more precise measures of adiposity, longitudinal designs, validated dietary assessments, and food-specific cognitive paradigms will be needed to clarify the mechanisms and clinical translation of these findings.

## Figures and Tables

**Table 1:** Participant characteristics (*M* ± *SD*).

Measure	Normal Weight	Overweight	Obese
*n*	21	22	19
Age (years)	20.2 ± 1.6	20.9 ± 1.6	19.8 ± 1.5
BMI (kg/m^2^) ^1^	22.2 ± 2.3	27.3 ± 1.2	33.5 ± 2.4
Height (cm)	168.8 ± 10.6	166.0 ± 9.6	164.6 ± 7.5
Weight (kg) ^1^	63.5 ± 10.4	75.3 ± 9.1	90.7 ± 8.1
VO_2_ Peak (mL/kg/min) ^1^	43.9 ± 9.9	38.0 ± 8.8	32.8 ± 6.2
IPAQ (MET-min/week)	2658.7 ± 1748.9	2634 ± 1514.9	1675.1 ± 963.5

^1^ Significant difference between normal weight, overweight, and obese participants, *p* < 0.05. BMI = body mass index; kg = kilogram; m = meter; cm = centimeter; mL = milliliter; min = minute; MET = metabolic equivalent.

**Table 2:** Summary of BMI–ERP associations across continuous, three-group, and median-split parameterizations.

ERP	Trial Type	Continuous BMI		Three-Group BMI			Median-Split BMI		
		*r*	*p*	*F*(2, 59)	*p*	*η* ^2^ *p*	*t*(60)	*d*	*p*
ERN	Overall	0.304	**0.016**	2.80	0.069	0.087	−2.72	−0.69	**0.009**
	Neutral	0.324	**0.010**	3.31	**0.043**	0.101	−1.99	−0.51	0.051
	Congruent	0.241	0.059	2.36	0.103	0.074	−1.78	−0.45	0.080
	Incongruent	0.302	**0.017**	2.31	0.108	0.073	−2.79	−0.71	**0.007**
Pe	Overall	−0.082	0.528	0.67	0.514	0.022	0.32	0.08	0.751
	Neutral	−0.087	0.504	0.37	0.693	0.012	0.23	0.06	0.819
	Congruent	−0.145	0.261	0.83	0.441	0.027	0.67	0.17	0.505
	Incongruent	−0.107	0.406	0.95	0.393	0.031	0.19	0.05	0.847

Statistically significant *p*-values (<0.05) are shown in **bold**. BMI = body mass index; ERN = error-related negativity; Pe = error positivity. Continuous BMI = Pearson correlation across the full sample. Three-Group BMI = one-way ANOVA across normal weight (*n* = 21), overweight (*n* = 22), and obese (*n* = 19) groups. Median-Split BMI = independent-samples t-test contrasting low-BMI (*n* = 31) and high-BMI (*n* = 31) groups, with negative *t* and *d* values indicating less negative ERN amplitude in the high-BMI group.

## Data Availability

The data presented in this study are available on request from the corresponding author. The data are not publicly available due to privacy and ethical reasons.

## Supplementary Materials

The following supporting information can be downloaded at: https://www.mdpi.com/article/10.3390/nu18152545/s1, Figure S1: Participant flow diagram; Table S1: Covariate-adjusted regression models predicting ERN and Pe amplitude; Table S2: Exploratory questionnaires–ERP correlations with FDR correction.

## Abbreviations

The following abbreviations are used in this manuscript:

EEG	Electroencephalography
EOG	Electrooculogram
ERP	Event-related potential
ERN	Error-related negativity
Pe	Error positivity
BMI	Body mass index
NHANES	National Health and Nutrition Examination Survey
PFC	Prefrontal cortex
ACC	Anterior cingulate cortex
M	Mean
SD	Standard deviation
ms	Milliseconds
kg	Kilogram
kΩ	Kilo-ohm
m	Meters
cm	Centimeters
mL	Milliliter
Hz	Hertz
μV	Microvolt
min	Minutes
K-BIT II	Kaufman Brief Intelligence Test, Second Edition
IPAQ	International Physical Activity Questionnaire
MET	Metabolic equivalent
MEQ	Mindful Eating Questionnaire
MAAS	Mindful Attention Awareness Scale
TFEQ-R18	Three-Factor Eating Questionnaire-Revised 18
BIS-11	Barratt Impulsiveness Scale, Version 11
UPPS-P	Urgency, Premeditation, Perseverance, Sensation Seeking, and Positive Urgency
I7Q	I7 Impulsiveness Questionnaire
SPSRQ	Sensitivity to Punishment and Sensitivity to Reward Questionnaire
VO2	Volume of Oxygen
EGI	Electrical Geodesics, Incorporated
IIR	Infinite impulse response
DC	Direct current
ICA	Independent component analysis
PCA	Principal Component Analysis
ROI	Region of interest
SPSS	Statistical Package for the Social Sciences
ANOVA	Analyses of variance
HSD	Honestly Significant Difference
ηp^2^	Partial eta-squared
α	Alpha
β	Beta
IQ	Intelligence quotient
DEXA	Dual-energy X-ray absorptiometry
MRI	Magnetic resonance imaging
IRB	Institutional Review Board
