# Supplementary material for: Body Mass Index and Dietary Self-Regulation Are Associated with Attenuated Error Monitoring in Young Adults
Source: Nutrients. 2026 Aug 4;18(15):2545. doi: 10.3390/nu18152545 (PMC13468073; doi:10.3390/nu18152545)
Supplement: Supplementary file 1 [file nutrients-18-02545-s001.zip › nutrients-4430940-supplementary.pdf]

Supplementary Materials

Figure S1. Participant Flow Diagram

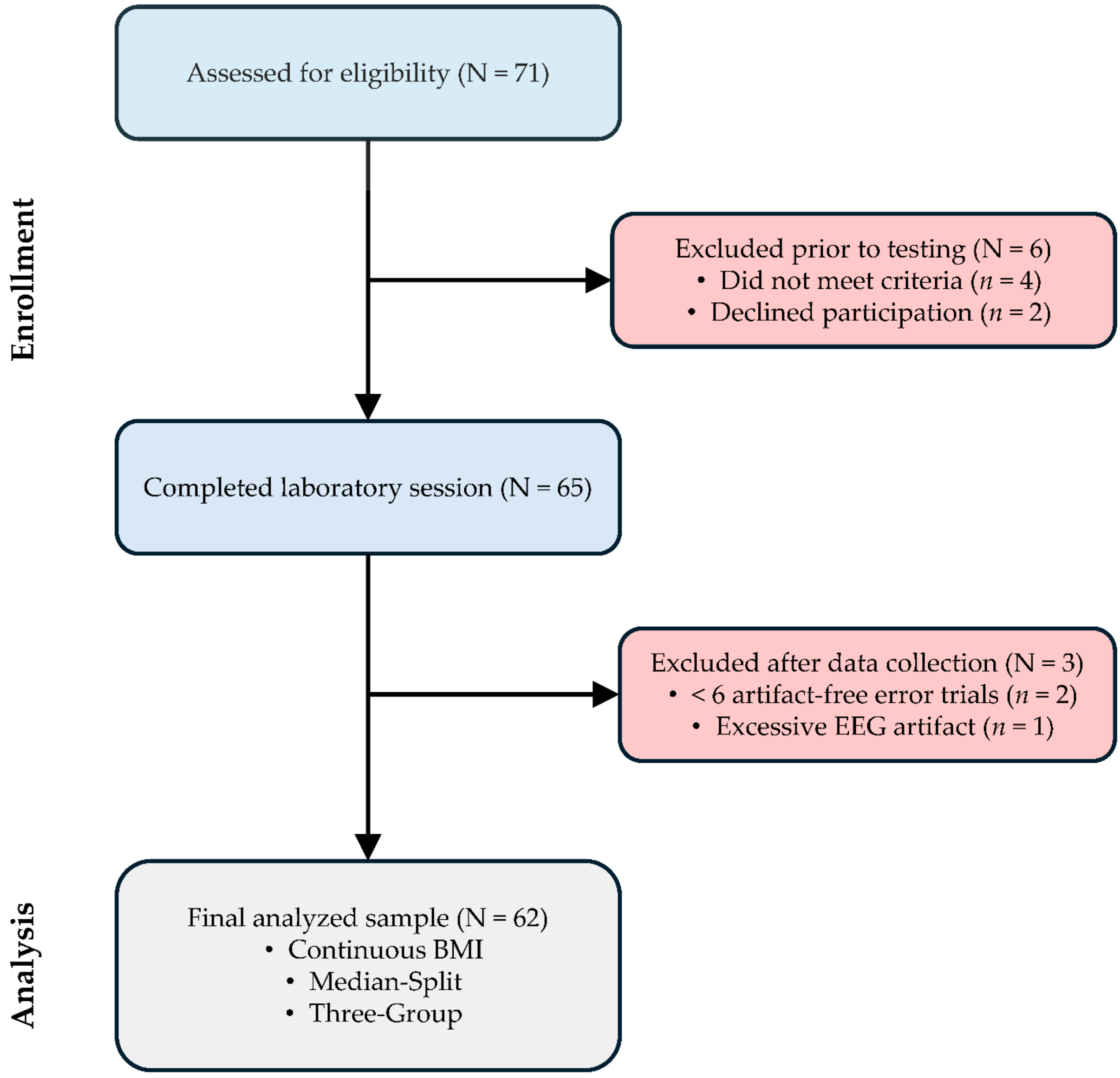

Table S1. Covariate-Adjusted Regression Models Predicting ERN and Pe Amplitude

Model: ERN (overall; N = 62; R^2 = 0.207, adjusted R^2 = 0.12).

Predictor	*b*	*SE*	*t*	*p*
BMI	**+0.072**	**0.031**	**+2.32**	**0.024**
Age	-0.049	0.084	-0.59	0.560
Sex	**-0.678**	**0.332**	**-2.04**	**0.046**
VO_2 peak	-0.002	0.020	-0.11	0.911
Physical activity (IPAQ)	-0.000	0.000	-0.11	0.913
Trait impulsivity (BIS-11 total)	-0.024	0.015	-1.60	0.115

Model: ERN (neutral; N = 62; R^2 = 0.182, adjusted R^2 = 0.093).

Predictor	*b*	*SE*	*t*	*p*
BMI	**+0.071**	**0.023**	**+3.05**	**0.004**
Age	+0.082	0.063	+1.31	0.197
Sex	-0.230	0.249	-0.92	0.360
VO_2 peak	+0.007	0.015	+0.46	0.647
Physical activity (IPAQ)	+0.000	0.000	+0.50	0.617
Trait impulsivity (BIS-11 total)	-0.018	0.011	-1.58	0.120

Model: Pe (overall; N = 62; R^2 = 0.03, adjusted R^2 = -0.076)

Predictor	*b*	*SE*	*t*	*p*
BMI	-0.078	0.101	-0.77	0.444
Age	+0.002	0.270	+0.01	0.995
Sex	-0.090	1.073	-0.08	0.933
VO_2 peak	-0.051	0.063	-0.81	0.419
Physical activity (IPAQ)	+0.000	0.000	+0.25	0.801
Trait impulsivity (BIS-11 total)	-0.026	0.048	-0.54	0.592

Statistically significant *p*-values (<.05) are shown in **bold**. BMI = body mass index. Sex coded 1 = male, 2 = female. Multiple linear regression models regressing each ERP amplitude on BMI and covariates (age, sex, VO_2 peak, physical activity, and trait impulsivity). Unstandardized coefficients (*b*), standard errors (*SE*), *t*, and *p*-values are reported.

Table S2. Exploratory Questionnaire–ERP Correlations with FDR Correction

Pe Amplitude

Questionnaire subscale	*r*	*p* (uncorrected)	*p* (FDR)
MEQ Disinhibition	0.35	0.005	0.101
UPPS-P Positive Urgency	-0.33	0.010	0.101
TFEQ Uncontrolled	-0.25	0.051	0.312
I7Q Empathy	0.24	0.059	0.312
I7Q Impulsiveness	-0.18	0.158	0.573
UPPS-P (lack of) Premeditation	-0.18	0.164	0.573
UPPS-P (lack of) Perseverance	-0.17	0.191	0.574
TFEQ Emotional	-0.16	0.229	0.601
SPSRQ Punishment	-0.14	0.295	0.622
MEQ Emotional	0.13	0.296	0.622
MEQ Distraction	-0.10	0.421	0.728
MAAS	0.10	0.425	0.728
BIS-11 Total	-0.10	0.457	0.728
UPPS-P Sensation Seeking	0.08	0.518	0.728
SPSRQ Reward	-0.08	0.520	0.728
UPPS-P Negative Urgency	-0.07	0.598	0.785
I7Q Venturesomeness	0.01	0.917	0.996
MEQ External	-0.01	0.918	0.996
IPAQ total MET-min	0.00	0.984	0.996
TFEQ Cognitive Restraint	0.00	0.988	0.996
MEQ Awareness	0.00	0.996	0.996

ERN Amplitude

Questionnaire subscale	*r*	*p* (uncorrected)	*p* (FDR)
MEQ Disinhibition	-0.35	0.005	0.100
UPPS-P Positive Urgency	-0.25	0.054	0.564
TFEQ Uncontrolled	0.18	0.161	0.785
I7Q Empathy	0.16	0.203	0.785
I7Q Impulsiveness	-0.15	0.232	0.785
UPPS-P (lack of) Premeditation	0.15	0.257	0.785
UPPS-P (lack of) Perseverance	-0.12	0.339	0.785
TFEQ Emotional	0.11	0.385	0.785
SPSRQ Punishment	-0.11	0.388	0.785
MEQ Emotional	-0.11	0.406	0.785
MEQ Distraction	-0.10	0.435	0.785
MAAS	-0.10	0.455	0.785
BIS-11 Total	-0.09	0.500	0.785
UPPS-P Sensation Seeking	-0.08	0.523	0.785
SPSRQ Reward	-0.05	0.675	0.935
UPPS-P Negative Urgency	0.03	0.822	0.935
I7Q Venturesomeness	-0.03	0.824	0.935
MEQ External	0.02	0.877	0.935

IPAQ total MET-min	-0.02	0.891	0.935
TFEQ Cognitive Restraint	0.01	0.918	0.935
MEQ Awareness	0.01	0.935	0.935

Pearson correlations between each questionnaire subscale and ERP amplitude (overall), with uncorrected *p*-values and Benjamini–Hochberg false discovery rate (FDR)-adjusted *p*-values computed within each ERP family. MEQ -= Mindful Eating Questionnaire; UPPS-P = Urgency, Premeditation, Perseverance, Sensation Seeking, and Positive Urgency; TFEQ = Three Factor Eating Questionnaire; I7Q = I7 Impulsiveness Scale; SPSRQ = Sensitivity to Punishment and Sensitivity to Reward Questionnaire; MAAS = Mindful Attention Awareness Scale; BIS-11 = Barratt Impulsiveness Scale; IPAQ = International Physical Activity Questionnaire. These analyses are exploratory and hypothesis-generating. Rows are ordered by uncorrected *p*-value.
